# ANO1 protein as a potential biomarker for esophageal cancer prognosis and precancerous lesion development prediction

**DOI:** 10.18632/oncotarget.8223

**Published:** 2016-03-21

**Authors:** Li Shang, Jia-Jie Hao, Xue-Ke Zhao, Jian-Zhong He, Zhi-Zhou Shi, Hui-Juan Liu, Li-Fei Wu, Yan-Yi Jiang, Feng Shi, Hai Yang, Yu Zhang, Yi-Zhen Liu, Tong-Tong Zhang, Xin Xu, Yan Cai, Xue-Mei Jia, Min Li, Qi-Min Zhan, En-Min Li, Li-Dong Wang, Wen-Qiang Wei, Ming-Rong Wang

**Affiliations:** ^1^ State Key Laboratory of Molecular Oncology, Cancer Institute (Hospital), Peking Union Medical College and Chinese Academy of Medical Sciences, Beijing 100021, China; ^2^ Department of Cancer Epidemiology, Cancer Institute (Hospital), Peking Union Medical College and Chinese Academy of Medical Sciences, Beijing 100021, China; ^3^ Henan Key Laboratory for Esophageal Cancer Research of the First Affiliated Hospital, Zhengzhou University, Zhengzhou 450000, China; ^4^ Department of Biochemistry and Molecular Biology, Shantou University Medical College, Shantou 515041, China; ^5^ Department of Histology and Embryology, Anhui Medical University, Hefei 230032, China; ^6^ Department of Gastroenterology, Anqing City Hospital, Affiliated Anqing Hospital of Anhui Medical University, Anqing 246003, China

**Keywords:** ANO1, esophageal squamous cell carcinoma, precancerous lesions, biomarker, prognosis

## Abstract

**Objectives:**

Anoctamin 1 (ANO1) has been found to be overexpressed in esophageal squamous cell carcinoma (ESCC) in our previous study. Herein we showed the clinical relevance of ANO1 alterations with ESCC and esophageal precancerous lesion progression.

**Results:**

ANO1 was detected in 38.1% (109/286) and 25.4% (77/303) of tumors in the two cohorts, but in none of morphologically normal operative margin tissues. ANO1 expression was significantly associated with a shorter overall survival (OS), especially in patients with moderately differentiated and stage IIA tumors. In 499 iodine-unstained biopsies from the endoscopic screening cohort in 2005-2007, all the 72 pathologically normal epithelial mucosa presented negative immunostaining, whereas ANO1 expression was observed in 3/11 tumors and 5/231 intraepithelial lesions. 7/8 ANO1-positive cases had developed unfavorable outcomes revealed by endoscopic follow-up in 2012. Analysis of another independent cohort of 148 intraepithelial lesions further confirmed the correlation between ANO1 expression and progression of precancerous lesions. 3/4 intraepithelial lesions with ANO1 expression had developed ESCC within 4-9 years after the initial endoscopic examination.

**Methods:**

Immunohistochemistry (IHC) was performed to examine ANO1 expression in surgical ESCC specimens and two independent cohorts of esophageal biopsies from endoscopic screening in high-incidence area of ESCC in northern China. Association between ANO1 expression, clinico-pathologic parameters, and the impact on overall survival was analyzed.

**Conclusions:**

Positive ANO1 is a promising biomarker to predict the unfavorable outcome for ESCC patients. More importantly, it can predict disease progression of precancerous lesions.

## INTRODUCTION

Esophageal carcinoma is one of the most common cancers. In China, squamous cell carcinoma (ESCC) is the predominant histological type of esophageal cancer. At the time of diagnosis, more than 50 percent of ESCC patients have unresectable tumors or radiographically visible metastases, and the overall five-year survival rate hovers around 10% for many years [1, 2]. Accurate diagnosis and classification are of vital importance for improving clinical outcomes. But up to date, no powerful markers have been identified to accurately classifying ESCC individuals in conjunction with therapeutics. Another gray area of clinical management involves precancerous lesions of the esophagus, which is divided into low-grade intraepithelial lesions (LSIL) and high-grade intraepithelial lesions (HSIL). LSIL includes mild dysplasia (mD) and moderate dysplasia (MD), and HSIL refers to severe dysplasia (SD) and carcinoma in situ (CIS). The clinical treatment for confirmed SD is usually the endoscopic mucosal resection, which is the same as that for CIS, but mD and MD are usually only regular follow-up [3]. Such management for precancerous lesions does not take into account the complex pattern of clonal evolution, which might lead to insufficient or excessive treatments. Identification of effective biomarkers that could be used to predict the prognosis of ESCC and the risk of precancerous lesions would be of great clinical benefits.

Anoctamin 1 (ANO1) has been recently identified to be overexpressed in esophageal carcinomas [4–7]. ANO1, also known as TMEM16A, ORAOV2, TAOS2, DOG1 or FLJ10261, is located on human chromosome 11q13. It contains 26 exons, encoding a 960 amino acid protein with eight transmembrane domains. ANO1 is associated with calcium-dependent chloride channel activity and involved in multiple biological functions including cell proliferation, motility and attachment [8, 9]. It has been shown that ANO1 contributes to the regulation of renal function, inflammatory and nerve-injury induced hypersensitivity [10]. Amplification and/or overexpression of ANO1 have been frequently observed in gastrointestinal stromal tumors (GISTs) [11–15], breast cancer, head and neck squamous cell carcinoma (HNSCCs) and gastric carcinomas [6, 8, 16] In ANO1-amplified cancer cell lines bearing 11q13 amplification, knockdown of ANO1 inhibited cell proliferation, induced apoptosis, and reduced tumor growth in established cancer xenografts via deactivating EGFR and CAMK signaling. Although a significant correlation has been found between ANO1 expression levels and overall survival (OS) of breast cancer patients [6], the clinical implication of ANO1 in human malignancies remain to be elucidated.

We previously found that ANO1 was amplified and overexpressed in both ESCC and esophageal dysplasia [7]. To further evaluate the clinical relevance of ANO1 alteration,  we  performed immunohistochemistry (IHC) to examine the expression of ANO1 protein not only in ESCCs who had undergone curative esophagectomy, but also in two independent biopsy cohorts of precancerous lesions to which we carried out an endoscopic follow-up for 4-9 years after their initial endoscopic examination.

## RESULTS

### ANO1 expression in ESCC tissues and association with clinico-pathological features

**Figure 1:**
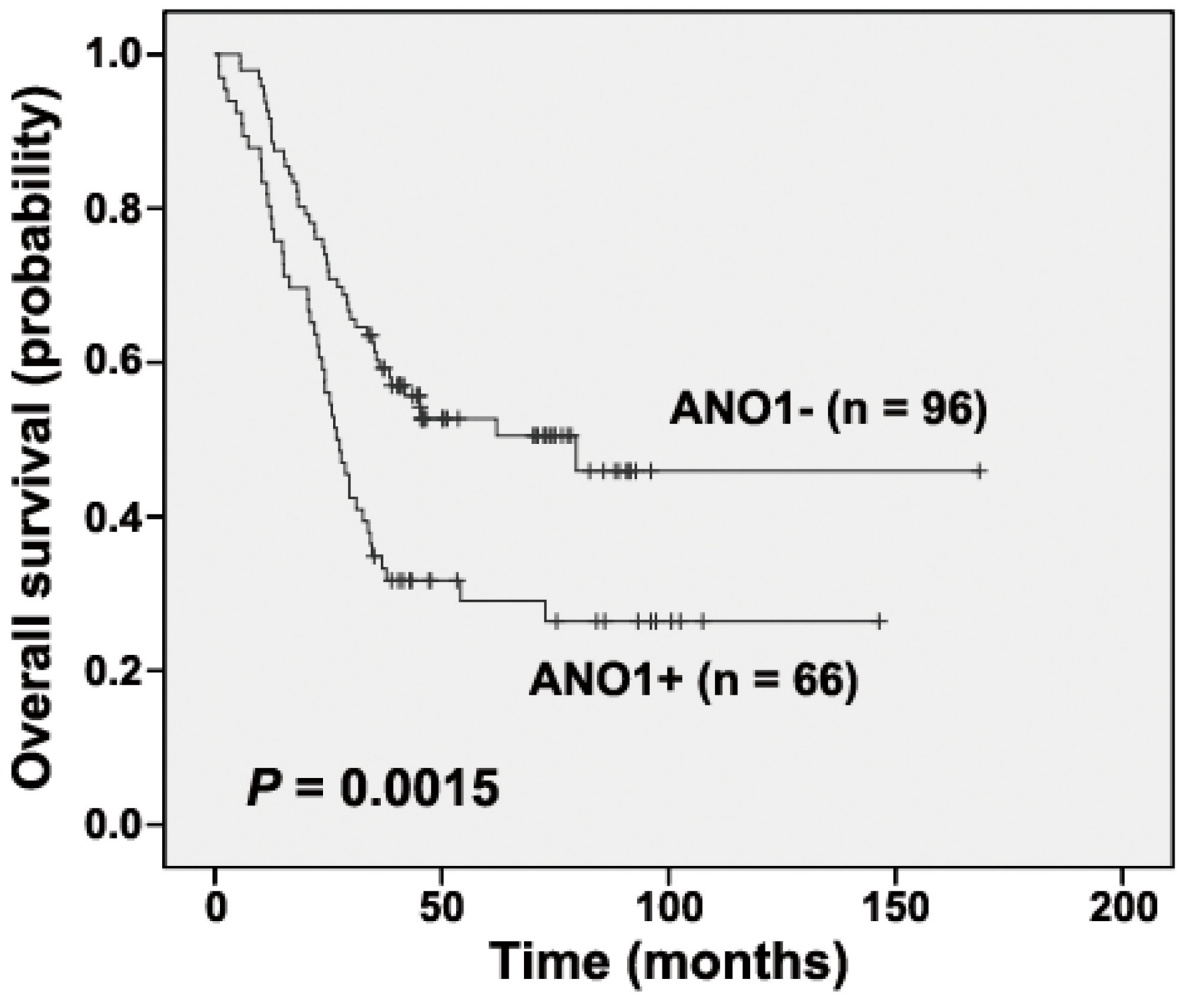
Kaplan-Meier analysis according to ANO1 status in cases from the first cohort (Log-rank test) “+”, positive; “-”, negative.

In the surgical specimens investigated, positive ANO1 staining was detected in 38.1% (109/286) of tumors from the first cohort, but in none of all the morphologically normal operative margin tissues (Supplementary Figure S1). ANO1 expression was seen in the cytoplasm and cell membrane of the tumor cells, and was significantly associated with the location of tumors (*P* = 0.0201, Supplementary Table S1). In the 286 tumors tested from the first cohort, the patient follow-up information of 162 cases was obtained, with an OS time of one to 168 months. Kaplan-Meier curves showed that patients with positive ANO1 expression had a shorter OS compared to those with undetectable expression (*P*= 0.0015, Figure 1). Univariate Cox proportional hazards regression analysis showed that lymph node metastasis, advanced tumor stages and ANO1 expression were significantly correlated with poorer OS (*P* = 0.0012, 0.0438 and 0.0018). Multiple Cox proportional hazards regression analysis indicated that lymph node metastasis and ANO1 expression were independent prognostic factors for ESCC patients (hazard ratio = 1.8234 and 1.9031, *P* = 0.0146 and 0.0020, Table 1).

**Table 1:** Univariate and multivariate cox regression analysis of overall survival of ESCC patients in CAMS cohort

Variable	Univariate analysis			Multivariate analysis		
	HR	95% CI	*P* value	HR	95% CI	*P* value
**Sex** (Female, Male)	1.2533	0.7767-2.0222	0.3550			
**Age** (≤ 60, > 60)	0.6990	0.4635-1.0542	0.0876			
**Macroscopic types** (Ul, Fu, Me)	1.0699	0.7665-1.4935	0.6912			
**Tumor location** (Up, Mi, Lo)	0.9403	0.6827-1.2950	0.7061			
**Tumor size** (≤ 5, > 5)	2.3524	0.8957-2.0419	0.1510			
**pT** (T1-2, T3-4)	1.3584	0.7046-2.6187	0.3604			
**pN** (N0, N1-3)	2.0212	1.3201-3.0947	0.0012	1.8234	1.1259-2.9528	0.0146
**Grade** (G1, G2, G3)	1.0777	0.7912-1.4679	0.6352			
**Stage** (I/IIA, IIB/IIIC)	1.9646	1.0188-3.7884	0.0438	1.3322	0.6337-2.8008	0.4493
**ANO1** (Neg, Pos)	1.9141	1.2729-2.8782	0.0018	1.9031	1.2653-2.8624	0.0020

HR, hazard ratio; CI, confidence interval; Ul, Ulcerative; Fu, Fungating; Me, Medullary; Up, Upper; Mi, Middle; Lo, Lower; pN, lymph node metastases; Neg: negative expression; Pos: positive expression.

**Figure 2:**
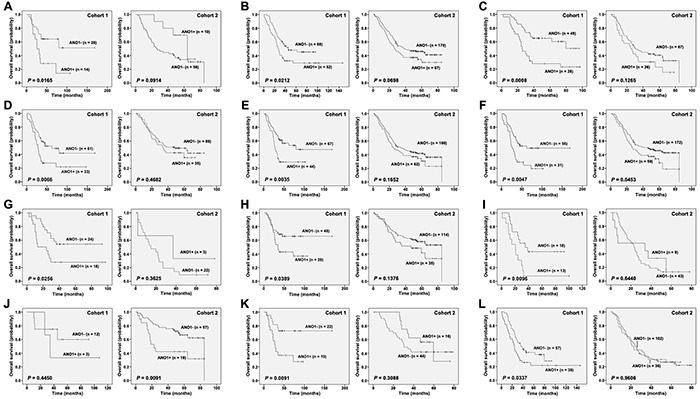
Stratified Kaplan-Meier analysis according to ANO1 status in two independent cohorts (Log-rank test) **A.** Female; **B.** Male; **C.** Age (> 60 years old); **D.** middle location; **E.** pT3; **F.** Grade 2; **G.** Grade 3; **H.** pN0; **I.** pN2; **J.** Stage IIA; **K.** Stage IIB; **L.** Stage III. “+”, positive; “-”, negative.

Stratified analysis in different clinico-pathological characteristics also indicated a poorer prognosis of ANO1-expressed patients, especially which were female (*P* = 0.0165), male (*P* = 0.0212), over 60 years old (*P* = 0.0008), and for those with tumors of middle location (*P* = 0.0066), moderate/poor differentiation (G2/3, *P* = 0.0047 and 0.0256, respectively), advanced stages (pT3 and stages IIB/III, *P* = 0.0035, 0.0091 and 0.0337, respectively), as well as without lymph node metastasis and with N2 (*P* = 0.0389 and 0.0096) (Table 2, Figure 2).

**Table 2:** Stratified analysis of ANO1 expression for overall survival in ESCC patients in two independent cohorts

Parameter	Cohort 1^#^		Cohort 2^#^	
	n	*P* value	n	*P* value
**Sex**				
Female	42	0.0165	66	0.0914
Male	120	0.0212	237	0.0698
**Age**				
≤ 60	85	0.2417	190	0.9951
> 60	77	0.0008	113	0.1265
**Tumor location**				
Upper	28	0.5970	18	0.5252
Middle	94	0.0066	124	0.4682
Lower	40	0.0544	161	0.3878
**pT**				
pT1	-	-	-	-
pT2	19	0.2484	48	0.1203
pT3	111	0.0035	242	0.1652
pT4	31	0.0623	-	-
**Grade**				
G1	33	0.8028	47	0.4760
G2	87	0.0047	231	0.0453
G3	42	0.0256	25	0.3625
**pN**				
N0	76	0.0389	149	0.1376
N1	43	0.5980	83	0.8570
N2	31	0.0096	52	0.6440
N3	12	0.7822	19	0.9773
**Stage**				
I	11	0.3112	29	0.7699
IIA	15	0.4450	76	0.0091
IIB	41	0.0091	60	0.3088
III	95	0.0337	138	0.9606

pT, pathologic T stage; pN, lymph node metastases.

Further stratified analysis was performed using another independent cohort from the second cohort and ANO1 were positively expressed in 25.4% (77/303) of patients. Also, all the morphologically normal operative margin tissues in this cohort showed ANO1 negative expression, which was the same as that in the first cohort. Although Kaplan-Meier curves showed no significant correlation between ANO1 expression and the OS of patients from the second cohort (*P* = 0.1615), patients with moderately differentiated and stage IIA tumors could be significantly stratified by ANO1 expression (*P* = 0.0453 and 0.0091) in the second cohort. Besides, male patients in both cohorts were stratified by ANO1 expression, although only marginal associations was observed in the second cohort (*P* = 0.0698) (Table 2, Figure 2).

### ANO1 expression in endoscopic biopsies in high-incidence areas

**Figure 3:**
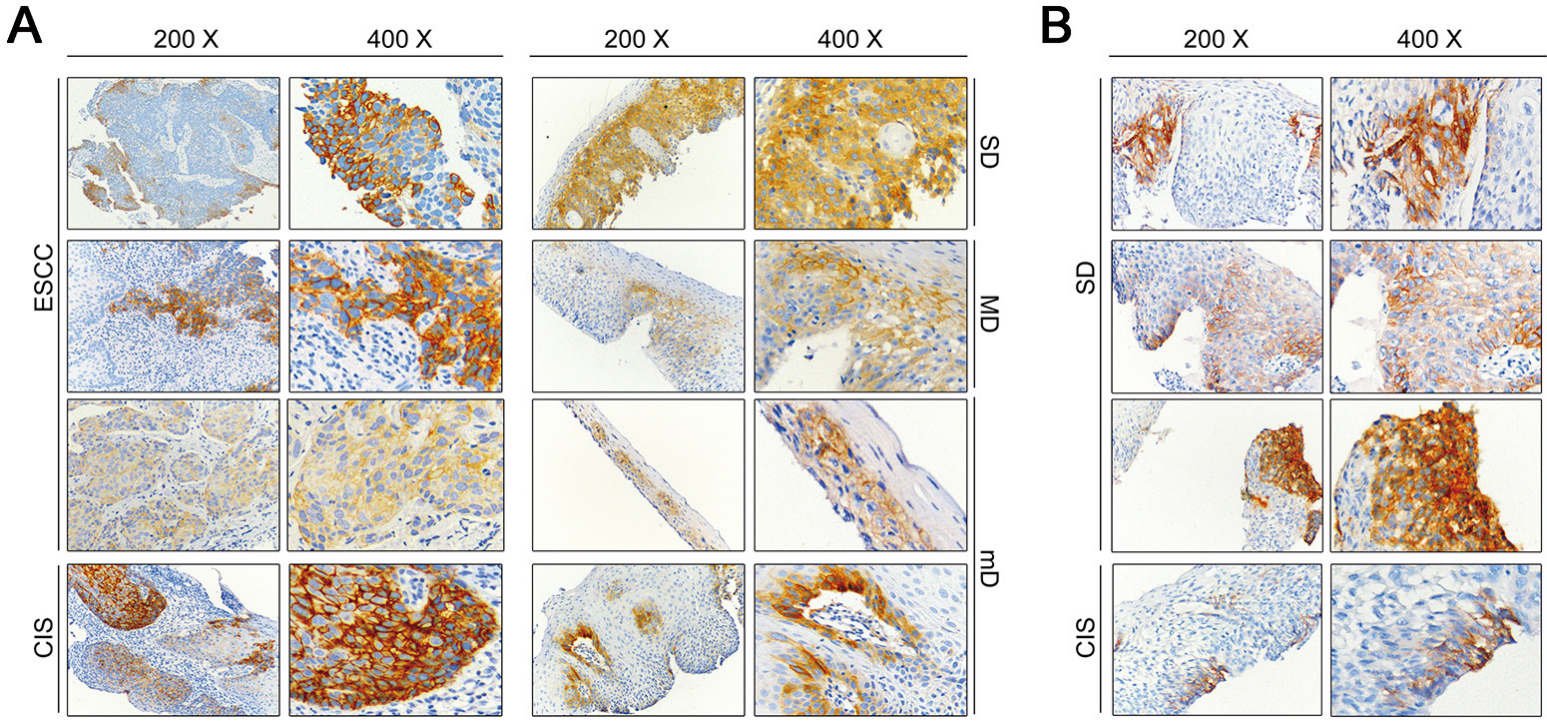
Representative immunohistochemical results of ANO1 positive staining **A.** Positive immunohistochemical staining of ANO1 in precancerous lesions and ESCC tissues in the sample set between 2005 and 2007 (magnification 200× and 400×). **B.** Positive immunohistochemical staining of ANO1 in precancerous lesions in the sample set between 1996 and 2002 (magnification 200× and 400×). mD, mild dysplasia; MD, moderate dysplasia; SD, severe hyperplasia; CIS, carcinoma in situ; ESCC, esophageal squamous cell carcinoma.

In the first cohort of biopsy specimens, ANO1 expression was detected in 3 out of 11 pathologically confirmed squamous carcinomas. Of the 416 cases with precancerous lesions, five cases presented positive ANO1 staining, including two cases with mD, one with MD, one with SD, and one with CIS (Figure 3A). However, all the 72 pathologically normal epithelial mucosa showed undetectable ANO1 immunoreaction. In the second cohort, ANO1 expression was observed in three of four cases with SD and one with CIS (Figure 3B), but undetectable in all the 43 mD, 6 MD and 94 chronic esophagitis.

### Progression of esophageal precancerous lesions with positive ANO1 expression in two independent cohorts from endoscopy screening

Endoscopic follow-up was carried out for 4-9 years after initial biopsy examination. The follow-up data of the two independent cohorts were summarized in Supplementary Tables S2–S3. We observed the progression of the cases with positive ANO1 expression. In the first cohort, all the three patients with squamous carcinomas had died at the follow-up time, those with SD or CIS developed squamous carcinomas, the case with MD progressed to intraepithelial carcinoma, one of the two mD progressed to MD and another regressed to a mild esophagitis. And in the second cohort, patients with CIS and two with SD had developed squamous carcinomas, and another SD remained unchanged (Table 3). Taken together, the precancerous lesions with positive ANO1 expression mostly had progressed in both the two cohort (80% and 100%), and the vast majority of those with negative ANO1 expression were no progression (87.8% and 100%) (Supplementary Table S4).

**Table 3:** Progression of esophageal lesions with positive ANO1 expression in two independent cohorts from endoscopy screening

Cohort	Initial examination	Initial diagnosis	Endpoint Time	Endpoint Result	Alteration
1st	2007	ESCC	2011	Died of ESCC	Progressed
1st	2007	ESCC	2012	Died of ESCC	Progressed
1st	2007	ESCC	2010	Died of ESCC	Progressed
1st	2006	CIS	2006	ESCC diagnosed	Progressed
1st	2007	SD	2012	ESCC diagnosed	Progressed
1st	2005	MD	2009	Intramucosal carcinoma	Progressed
1st	2007	mD	2012	MD diagnosed	Progressed
1st	2005	mD	2012	ES diagnosed	Regressed
2nd	2001	CIS	2005	ESCC diagnosed	Progressed
2nd	1998	SD	2000	ESCC diagnosed	Progressed
2nd	1996	SD	2011	Died of ESCC	Progressed
2nd	2000	SD	2005	SD diagnosed	Unchanged

ES, esophagitis; mD, mild dysplasia; MD, moderate dysplasia; SD, severe hyperplasia; CIS, carcinoma in situ; ESCC, esophageal squamous cell carcinoma.

## DISCUSSION

In the present study, we demonstrated that ANO1 expression was an independent prognostic biomarker for shorter survival of ESCC patients. More importantly, we showed for the first time that ANO1 expression was a potential biomarker for predicting disease progression in subjects with esophageal precancerous lesions.

We observed that ANO1 expression was positive in 38.1% (109/286) and 25.4% (77/303) of primary esophageal tumors from the two independent cohorts, respectively. Excitingly, all the morphologically normal operative margin tissues from both cohorts showed negative ANO1 expression. Also, in the endoscopic biopsies, all the pathologically normal epithelial mucosa and chronic esophagitis showed undetectable ANO1 immunoreaction. These results suggest a high specificity (100%) of ANO1 expression.

We found a correlation between ANO1 expression and shorter survival time of patients. Multiple Cox regression identified that ANO1 expression was an independent prognostic factor for shorter survival. When comparing the stratified analysis results from two independent cohorts, we found that ANO1-expressed patients with moderately differentiated tumors showed poorer prognosis in both cohorts. It is noted that two different prognosis groups could also be separated according to Kaplan-Meier curves when stratification analysis was performed on patients that were male and over 60 years old, and those with pT3 and pN0 tumors, although the differences were not significant in the second cohort. Besides, stage IIA patients were stratified by ANO1 expression in the second cohort but not in the first cohort. Whereas middle location, poor differentiation, pN2, stages IIB/III could significantly stratify patients in the first cohort but not in the second cohort. The possible reason for the discrepancies among different cohorts is that the patterns of other molecular alterations may be variable among different areas, in which populations, environments and carcinogenic factors may be different from each other. Whether it is caused by the area differences should be further validated. But importantly, we noted that in tumors of ANO1 immunostaining score ≥ 6 in the present study, 53.1% (26/49) had died up to the follow-up time, in which primary tumors of eleven patients were at stages I-II. These data suggest that ANO1 expression could indicate a subset of patients for whom more aggressive treatments and close follow-up should be considered.

Based on that ANO1 expression presented 100% specificity in morphologically normal operative margin tissues and its prognostic implication in patients with advanced ESCC, we further investigated ANO1 alteration in esophageal precancerous lesions according to the follow-up information. We observed that 80% (four out of five) of ANO1-positive cases detected in initial biopsies showed lesion progression at the follow-up time in the first cohort, and 75% (three out of four) showed progression in the second cohort. Of these seven progressed cases, five patients had developed invasive squamous carcinomas, one progressed to intramucosal carcinoma, and one mD progressed to MD. The data indicate that ANO1 may be a potential biomarker for predicting malignant progression of precancerous lesions. In China, the overall survival rate of ESCC patients is less than 10%, but as high as 85% if the patients are treated at an earlier stage [2, 17]. A previous study showed that endoscopic mucosal resection (EMR) had an excellent long-term survival rate of 97% with acceptable function and quality of life [18]. Therefore, the development of appropriate early detection and prediction biomarkers for curable lesions offer a great opportunity to reduce the risk of progressing to ESCC. Our results suggest that mucosal resection plus a strengthened follow-up might be essential for ANO1-positive esophageal precancerous lesions, especially for those with strong ANO1 expression. In the future, we will extend the present study to a prospective investigation in order to confirm the potential clinical value of ANO1 for predicting the progression of precancerous lesions.

It has been revealed that ANO1-induced cancer cell proliferation was accompanied by an increase of extracellular signal–regulated kinase (ERK)1/2 activation and cyclin D1 induction in head and neck squamous cell carcinoma. Pharmacologic inhibition of MEK/ERK and genetic inactivation of ERK1/2 could abrogate the growth effect of ANO1 [19]. A similar ANO1-regulated ERK1/2 phsphorylation has been observed in mouse ovarian granulosa cells [20]. We previously reported that knock-down of ANO1 significantly inhibited the proliferation of KYSE30 and KYSE510 cells [7]. However, the mechanisms underlying the involvement of ANO1 in esophageal carcinogenesis remains unclear, to which further investigation should be addressed.

In conclusion, ANO1 expression can serve as a poorer prognosis biomarker for ESCC patients to whom a more aggressive treatment should be considered. Especially, our study provides an important candidate biomarker to predict disease progression in individuals with esophageal precancerous lesions.

## MATERIALS AND METHODS

### Patients and samples

A total of 589 surgically resected primary ESCC tissues of two independent cohorts from two different areas of China and 647 esophageal endoscopic biopsies (including 564 intraepithelial lesions) were used in the present study.

286 ESCC tissues from the first cohort were collected between 1998 and 2008 at the Cancer Hospital, Chinese Academy of Medical Sciences and Peking Union Medical College (CAMS & PUMC) in Beijing, China. 303 ESCC tissues from the second cohort were collected between 2007 and 2011 at Medical College of Shantou University in Shantou, China. All the operative samples were residual specimens after diagnostic sampling. All the patients received no treatment before surgery, and patients who died within one month after surgery were excluded. Clinico-pathological characteristics of the 589 ESCC patients were summarized in Supplementary Table S5.

The biopsy tissues included two independent cohorts. The first one contained 499 iodine-unstained biopsies collected by endoscopic screening from 2005 to 2007 in ESCC high-incidence area, Linxian and Cixian, China. Among them, 72 were pathologically diagnosed as normal epithelia, 11 as squamous carcinomas and 416 as precancerous lesions (339 mD, 54 MD, 19 SD and 4 carcinoma in situ). The second cohort included 94 chronic esophagitis tissues and 54 precancerous lesions (43 mD, 6 MD, 4 SD and one carcinoma in situ) procured by endoscopic examination in another rural area of Linxian from 1996 to 2002. All the biopsy specimens for this study were collected as residual tissue sections after clinical diagnosis. Clinico-pathological characteristics of the 564 (416+94+54) patients with esophageal precancerous lesions were summarized in Supplementary Table S6. For patients of the two cohorts, endoscopic follow-up was carried out in 2012 for the first cohort and 4-9 years after initial endoscopic examination for the second cohort.

Every patient signed separate informed consent forms for sampling and molecular analysis. Tissues were routinely formalin-fixed and paraffin-embedded.

This study was approved by the Ethics Committee/Institutional Review Board of the Cancer Institute (Hospital), Peking Union Medical College and Chinese Academy of Medical Sciences (No. 12-097/631).

### Sample preparation and IHC procedure

Tissue microarrays (TMAs) for advanced carcinomas were constructed as described previously [21], and the resulting blocks were cut into 4-μm sections. Biopsy tissues were cut into 4-μm full sections. The slides were deparaffinized, rehydrated, immersed in 3% hydrogen peroxide solution for 10 min, heated in citrate buffer (pH 6.0) for 25 min at 95¼C, and cooled for 60 min at room temperature. Between each incubation step, the slides were washed with phosphate buffered saline (PBS, pH 7.4). Then the slides with TMAs were incubated with rabbit monoclonal antibody (1:50 dilution, LifeSpan BioSciences, Seattle, USA) overnight at 4¼C. Immunostaining was performed using the PV-9000 Polymer Detection System with diaminobenzidine (DAB) according to manufacturer recommendations (GBI, USA) and subsequently counterstained with hematoxylin. Slides without the addition of primary antibody served as negative control.

### Assessment of IHC results

Assessment and imaging of the IHC results were performed using a Leica DM2000 microscope equipped with Leica DFC Cameras-Image Acquisition System (software V3.5.0, Switzerland). Immunohistochemical staining was scored blindly with no information on the clinical data provided. ANO1 protein expression was determined based on staining intensity and the percentage of immunoreactive cells. The staining intensity was rated as 0 (no staining), 1 (weak staining), 2 (moderate staining), or 3 (strong staining). The percentage of immunoreactive cells was graded as 0 (≤ 10%), 1 (11-25%), 2 (26-50%), or 3 (> 50%). Tissue IHC score was calculated by multiplying the intensity and the percentage of positive tumor cells.

### Statistical analysis

Statistical analysis was performed with the SPSS software program (version 17.0). Chi square test was used to assess the relationship between ANO1 expression and clinico-pathological parameters. Kaplan-Meier survival curves were constructed, and the differences were detected by the Log-rank test. The clinical end point was OS, defined as time from surgery to death. The data of patients alive at the end of the study were censored. Multiple Cox proportional hazards regression was carried out to identify the independent factors with a significant impact on patient survival. *P* values less than 0.05 were considered statistically significant.

## SUPPLEMENTARY FIGURE AND TABLES


