# Supplementary material for: ANO1 protein as a potential biomarker for esophageal cancer prognosis and precancerous lesion development prediction
Source: Oncotarget. 2016 Mar 21;7(17):24374–82. doi: 10.18632/oncotarget.8223 (PMC5029708; doi:10.18632/oncotarget.8223)
Supplement: Supplementary file 1 [file oncotarget-07-24374-s001.pdf]

SUPPLEMENTARY FIGURE AND TABLES

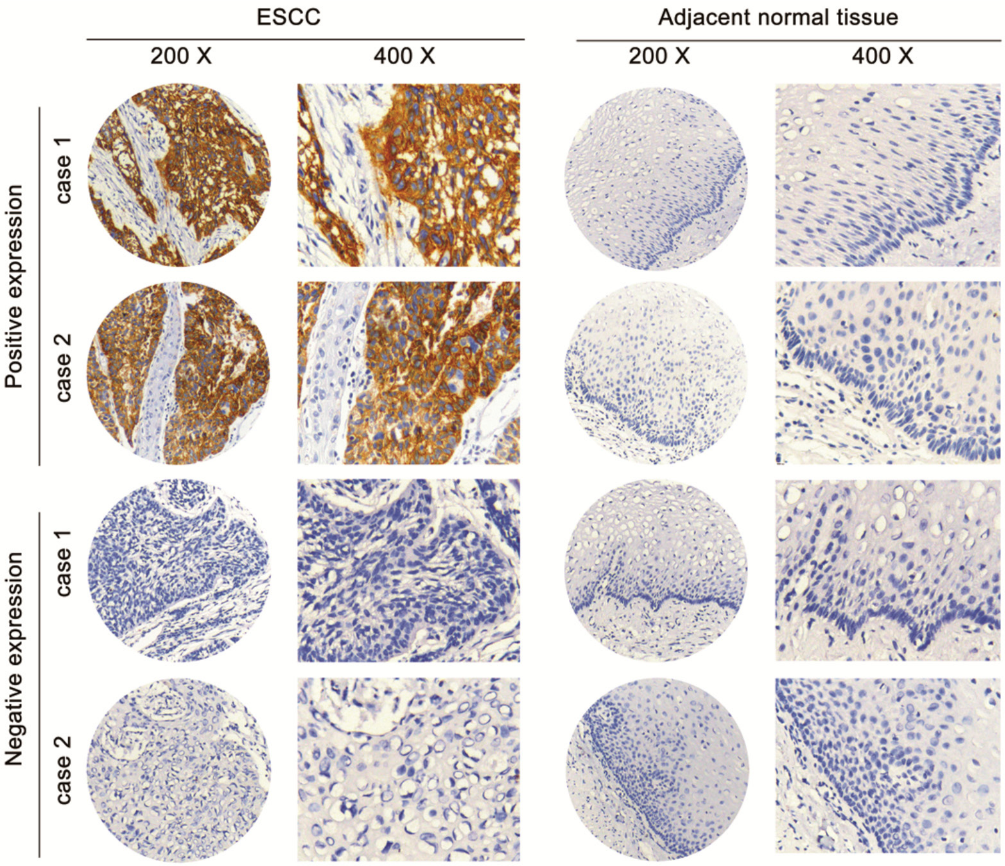

Supplementary Figure S1: Representative immunohistochemical results detected in ESCC and morphologically normal operative margin tissues (magnification 200× and 400×). Top two rows on the left column, high ANO1 expression, and bottom two rows, negative ANO1 in tumors; Right column, no expression in operative margin tissues. ESCC, esophageal squamous cell carcinoma; Normal, morphologically normal operative margin tissues.

Supplementary Table S1: Relationship between positive expression of ANO1 and clinico-pathological parameters in CAMS cohort

Parameter	ANO1 expression	*P* value
**Sex**		
Female	23/70 (32.9%)	0.2976
Male	86/216 (39.8%)	
**Age at diagnosis (year)**		
≤ 60	59/144 (41.0%)	0.3159
> 60	50/142 (35.2%)	
**Macroscopic type**		
Ulcerative	8/19 (42.1%)	0.7231
Fungating	37/88 (42.0%)	
Medullary	63/175 (36.0%)	
Others	1/4 (25.0%)	
**Tumor location**		
Upper	20/45 (44.4%)	0.0201
Middle	59/182 (32.4%)	
Lower	30/58 (51.7%)	
**Tumor size (cm)**		
≤ 5	52/143 (36.4%)	0.5468
> 5	55/138 (39.9%)	
**Histology grade**		
G1	24/62 (38.7%)	0.9063
G2	58/156 (37.2%)	
G3	22/63 (34.9%)	
**pT**		
T1	0/2 (0.0%)	0.1970
T2	12/26 (46.2%)	
T3	77/190 (40.5%)	
T4	20/68 (29.4%)	
**pN**		
N0	52/135 (38.5%)	0.8951
N1	30/80 (37.5%)	
N2	16/46 (34.8%)	
N3	11/25 (44.0%)	
**Stage**		
I	8/16 (50.0%)	0.3087
IIA	8/27 (29.6%)	
IIB	31/69 (44.9%)	
III	62/174 (35.6%)	

G1, well differentiated; G2, moderately differentiated; G3, poorly differentiated; pT, pathologic T stage; pN, lymph node metastases.

Supplementary Table S2: The follow-up data of the first cohort

The first cohort	Progressed-free	Progressed	Total number
mD	301	38	339
MD	50	4	54
SD	11	8	19
CIS	0	4	4
Total number	362	54	416
	**Survival after treatment**	**Progressed/died**	
Intramucosal carcinoma	0	4	4
Invasive carcinoma	3	4	7
Total number			427

Abbreviations: mD=mild dysplasia; MD=moderate dysplasia; SD=severe hyperplasia; CIS=carcinoma in situ.

Supplementary Table S3: The follow-up data of the second cohort

The second cohort	Progressed-free	Progressed	Total number
ES	91	3	94
mD	42	1	43
MD	6	0	6
SD	2	2	4
CIS	0	1	1
Total number	141	7	148

Abbreviations: ES=oesophagitis; mD=mild dysplasia; MD=moderate dysplasia; SD=severe hyperplasia; CIS=carcinoma in situ.

Supplementary Table S4: The comparison of different progression of precancerous patients with positive and negative ANO1 expression in the two cohort

	Cohort 1[#]		Cohort 2[#]	
	**Progressed** N = 54	**Progressed-free** N = 362	**Progressed** N = 4	**Progressed-free** N = 50
**ANO1(+)**	4 (80%)	1 (20%)	4 (100%)	0 (0%)
**ANO1(-)**	50 (12%)	361 (88%)	0 (0%)	50 (100%)

Abbreviations: +: positive expression; -: negative expression.

Supplementary Table S5: Clinico-pathological characteristics of 589 ESCC patients from two areas of China

Characteristics	Case (%)	
	Cohort 1# (n = 286)	Cohort 2# (n = 303)
**Sex**		
Female	70 (24.5)	66 (21.8)
Male	216 (75.5)	237 (78.2)
**Age at diagnosis (year)**		
≤ 60	144 (50.3)	190 (62.7)
> 60	142 (49.7)	113 (37.3)
**Macroscopic type**		
Ulcerative	19 (6.6)	
Fungating	88 (30.8)	
Medullary	175 (61.2)	
Others	4 (1.4)	
**Tumor location**		
Upper	45 (15.8)	18 (5.9)
Middle	182 (63.9)	124 (40.9)
Lower	58 (20.4)	161 (53.1)
**Tumor size, cm**		
≤ 5	143 (50.9)	
> 5	138 (49.1)	
**Histology grade**		
G1	62 (22.1)	47 (15.5)
G2	156 (55.5)	231 (76.2)
G3	63 (22.4)	25 (8.3)
**pT**		
Tis/T1	2 (0.7)	12 (4.0)
T2	26 (9.1)	48 (15.8)
T3	190 (66.4)	242 (79.9)
T4	68 (23.8)	1 (0.3)
**pN**		
N0	135 (47.2)	149 (49.2)
N1	80 (28.0)	83 (27.4)
N2	46 (16.1)	52 (17.2)
N3	25 (8.7)	19 (6.3)
**Stage**		
I	16 (5.6)	29 (9.6)
IIA	27 (9.4)	76 (25.1)
IIB	69 (24.1)	60 (19.8)
III	174 (60.8)	138 (45.5)

G1, well differentiated; G2, moderately differentiated; G3, poorly differentiated; pT, pathologic T stage; pN, lymph node metastases.

Supplementary Table S6: Clinico-pathological characteristics of 564 endoscopic biopsies with esophageal precancerous lesions in two independent cohorts

Characteristics	Cohort 1# (n = 416) Positive case Nb. (%)	Cohort 2# (n = 148) Positive case Nb. (%)
**Sex**		
Female	249 (59.9)	60 (40.5)
Male	167 (40.1)	88 (59.5)
**Age at diagnosis (year)**		
< 40	0 (0.0)	36 (24.3)
40-50	142 (34.1)	59 (39.9)
51-60	218 (52.4)	35 (23.6)
61-70	56 (13.5)	18 (12.2)
**Location**		
Upper	37 (8.9)	0 (0.0)
Middle	197 (47.5)	85 (57.4)
Lower	181 (43.6)	63 (42.6)
**Pathologic diagnosis**		
ES	0 (0.0)	94 (63.5)
mD	339 (81.5)	43 (29.0)
MD	54 (13.0)	6 (4.1)
SD/CIS	23 (5.5)	5 (3.4)

ES, esophagitis; mD, mild dysplasia; MD, moderate dysplasia; SD, severe hyperplasia; CIS, carcinoma in situ.
